# Ten simple rules for socially responsible science

**DOI:** 10.1371/journal.pcbi.1010954

**Published:** 2023-03-23

**Authors:** Alon Zivony, Rasha Kardosh, Liadh Timmins, Niv Reggev

**Affiliations:** 1 Department of Psychological Sciences, Birkbeck College, University of London, London, United Kingdom; 2 Department of Psychology, University of Sheffield, Sheffield, United Kingdom; 3 Department of Psychology, New York University, New York, United States of America; 4 School of Psychology, Faculty of Medicine, Health and Life Sciences, Swansea University, Swansea, Wales, United Kingdom; 5 Department of Psychology, Ben-Gurion University of the Negev, Be’er-Sheva, Israel; 6 School of Brain Sciences and Cognition, Ben-Gurion University of the Negev, Be’er-Sheva, Israel; Carnegie Mellon University, UNITED STATES

## Abstract

Guidelines concerning the potentially harmful effects of scientific studies have historically focused on ethical considerations for minimizing risk for participants. However, studies can also indirectly inflict harm on individuals and social groups through how they are designed, reported, and disseminated. As evidenced by recent criticisms and retractions of high-profile studies dealing with a wide variety of social issues, there is a scarcity of resources and guidance on how one can conduct research in a socially responsible manner. As such, even motivated researchers might publish work that has negative social impacts due to a lack of awareness. To address this, we propose 10 simple rules for researchers who wish to conduct socially responsible science. These rules, which cover major considerations throughout the life cycle of a study from inception to dissemination, are not aimed as a prescriptive list or a deterministic code of conduct. Rather, they are meant to help motivated scientists to reflect on their social responsibility as researchers and actively engage with the potential social impact of their research.

This is a *PLOS Computational Biology* Methods paper.

More than ever before, scientists are being called upon to acknowledge and engage with the social impact of their scientific outputs (see [1–3] for reviews). This is perhaps most clearly reflected in a paper about racial disparities in police shootings in the United States [4]. The authors of this study reported that they found “no evidence of anti-Black or anti-Hispanic disparities.” Following heavy criticism [5,6], the study was subsequently retracted, not by the journal but rather by the authors themselves. The authors initially rejected the scientific criticism [7]. However, they later justified their retraction due to “the continued use” of the work to “support for the idea that there are no racial biases in fatal shootings, or policing in general” [8]. In other words, scientists concluded that they should retract their study in the name of social responsibility, as it was written in a way that could have harmful effects (“grimpacts;” [9,10]) on public discourse (and thus potentially harm specific groups).

This study is but a single example out of a string of recent high-profile studies from various fields that drew harsh criticism from the general public and scientists alike for using science to promote ideas that could potentially inflict harm on individuals and social groups (e.g., [4,11–21]). Such concerns are far from new (e.g., [22–26]). However, although scientific papers used to be accessible only to relatively few experts, such outputs can nowadays reach an incredibly wide audience very quickly. A study that captures the public’s attention can reach an audience of many millions via online news outlets, Twitter, podcasts, TV shows, YouTube channels, online forums, and so on. This puts great power over the public sphere in the hands of relatively few scientists. Moreover, the speed and breadth of dissemination expose scientists to a new challenge that may catch many of us off-guard: addressing the myriad ways our published research will be interpreted and evaluated by the general public.

The recent torrent of visible yet contentious studies points to the difficult question: What is the responsibility of scientists over the social impacts of their research? This question has been debated for over a century. Not long ago, many scientists rejected any responsibility for social impacts, as such responsibility was viewed to be in direct contradiction with scientific freedom (see [1] for review). In the last few decades, this attitude has been slowly changing, and a consensus has been growing that scientists should be responsible for at least some consequences of their research. For example, it is now widely agreed that scientists should minimize the potential risks to the physical and mental well-being of people and populations participating in their studies. Indeed, such considerations have been institutionalized and are regulated via education, periodic training, and regulation by ethics boards all over the world [27]. Moreover, growing concerns about the societal ramifications of emerging technologies have already led to substantial policy changes among many actors and stakeholders involved in science and technology development (e.g., [3,28]). This is perhaps most clearly exemplified by the recent push for policies that promote “responsible development” or “Responsible Research and Innovation”—research processes that account for sustainability and potential impacts on society and aim to produce “socially desirable” outcomes [2].

Given these changes, the time seems ripe for scientists to consider their responsibility over the possible impact of their research outputs. However, determining the desired degree of individual responsibility involves significant challenges, as scientists can only be asked to have responsibility for impacts that are “reasonably” foreseeable [1,10]. In many cases, research can have indirect social impacts that are impossible to predict. For example, a study that focuses on a specific social group (see examples below) can help shape the general public’s beliefs about the said group. While it is widely agreed that social beliefs can have real effects on the physical well-being, psychological welfare, and livelihood of people (e.g., [29–32]), it is hard to tell what effect, if any, can be attributed to the specific study in question [33]. In such indirect cases, what is considered a “reasonably foreseeable impact” will often be a matter of debate.

It is also arguable that promoting socially responsible science should rely on institutionalization and regulation by scientific organizations rather than thrusting more responsibilities upon individual scientists. Policy and structural factors, rather than individual actions, are viewed as the key to ensuring responsibility and societal desirability of the scientific process and its outputs [2,3,34,35]. Indeed, without relevant education, clearly articulated and regulated standards, and support in their interpretation and implementation, it is unlikely that already overburdened individual scientists will successfully integrate all ethical considerations in their research. Unfortunately, such structures are largely absent in many scientific fields. For example, in many fields, scientists receive some training in the ethical treatment of human participants or animal subjects (e.g., [36]) but little to no training in considering the ethical ramifications of their work on society (with a few notable exceptions, such as several subdisciplines of sociology and anthropology, e.g., [37–39]).

Moreover, scientists are actually incentivized to disregard any aspects of their work that hinder swift publication, such as addressing the limitations of their methods or considering the potential long-term, broad implications and interpretations of their results. In this climate, current scientific structures discourage social responsibility. The competition for jobs and funding opportunities in academia drives scientists to churn out high-impact publications at an ever-increasing rate [40,41]. Consequently, to maximize impact, scientists are incentivized to publish novel or controversial findings while overstating the veracity of their conclusions [42–44]. In short, scientists are pushed to vie for the public’s attention but to downplay or ignore altogether any negative social impact (“grimpacts”) their research might have [9].

Given these constraints, formally—and justly—characterizing scientists’ obligations to minimize the potential societal harm of their research remains a daunting task, especially when such harm is indirect. When it comes to broad societal impacts, it may take a long time before the scientific community can agree on how to balance scientific freedom on the one hand and the principles of benevolence and non-maleficence in the context of broad societal impacts on the other (for a related discussion, see [45]). Until such time comes, we would like to offer a potential path forward.

In this paper, we offer 10 simple rules for socially responsible science. We follow the life cycle of a study, from inception to dissemination, and provide concrete suggestions that can help scientists to reflect, plan, and act to minimize potential societal harms stemming from their work. Because different scientists may consider social impacts at different stages, and because the production of scientific output is far from linear, these rules overlap to some degree. Undoubtedly, these rules cannot replace a broader structural shift in how science is done. However, in the absence of structural support and education, even researchers who wish to be socially responsible might publish work that has negative impacts due to a lack of awareness (for a similar point, see [46,47]). The following list of rules is meant for these scientists. We emphasize that our purpose is not to provide a prescriptive list against which any individual study or scientist can be evaluated, nor do we propose this list as a fixed and deterministic code of conduct. Rather, we aim to highlight straightforward considerations in order to empower individual scientists to actively engage with the potential social impact of their research (even in cases where such impact is indirect). Moreover, we acknowledge that in drafting these rules, we drew on our own experiences and paradigms; therefore, this list cannot be entirely comprehensive. Nevertheless, we hope this list can enrich the conversation about individual and collective social responsibility in science that is sorely missing outside a few select fields. At the very least, we hope that these rules can help scientists to avoid unwittingly causing harm to others and help them to navigate potential criticisms from the general public and other scientists.

## Rule 1: Get diverse perspectives early on

Science is inherently collaborative. We pool our expertise to work together on projects and depend on knowledgeable peers to critically evaluate our ideas and research. Peers from other fields or peers with knowledge we do not have are particularly helpful in this regard. Without them, we run the risk of overestimating how well-informed we are [47–49]. Similarly, when studying topics related to a particular marginalized group, we can greatly benefit from reaching out to members of said group, as they may have valuable, highly accurate [50] “insider” knowledge [51,52], based on their experiences. To varying degrees, some fields recognize the benefits of “participatory research” [53] and of viewing the community in question as a research partner [54] (e.g., qualitative research, public health). Unfortunately, however, in many other empirical sciences, these insights are almost entirely absent. When we view a social group as a research topic rather than an equal partner and ignore our own limited knowledge, we risk creating flawed designs and introducing easily avoidable errors. For example, a study that aimed to examine the genital arousal patterns of bisexual men in response to erotic stimuli supposedly found no difference between the attraction pattern of self-identified bisexual men and gay men [55]. Following this study, news outlets such as the New York Times published articles that called into question the very existence of bisexuality in men, proclaiming that bisexual men are either “Straight, Gay or Lying.” Aside from other criticisms about this line of research and what can be learned from it (e.g., [56,57]), a later study informed by consultation with representatives from the bisexual community found that the original result was merely an artifact of inadequate sampling and screening [58]. Had the researchers consulted with the community of interest early on, the negative impact of doubts cast on bisexual men and portrayals of bisexual men as untrustworthy would have been avoided (see [59] for a similar conclusion regarding flawed research into d/Deaf signing communities).

Recent efforts to adopt an inclusive approach to studying diverse populations span multiple disciplines and content, such as race [60], autism [61,62], artificial intelligence [63,64], and pedagogy [65]. We follow suit and recommend that scientists should try to get inclusive perspectives on their work by identifying the populations impacted by their study and engaging them at the earliest stages [47]. Efforts are being made to do this in multiple disciplines and localities. For example, Community-Based Participatory Research in North America and Patient and Public Involvement in the UK are 2 commendable initiatives to involve lay members of the public to contribute and collaborate on research that affects their lives [66,67]. In addition to taking such approaches, we can invite insider researchers to be our coauthors or to consult on our work with adequate compensation. Importantly, such collaborations should not be cursory. When in the position of being the “outsider” researchers working with insiders (whether fellow researchers or members of the public), we should be ready and willing to share power and control over a given project with those who will be most affected by its findings.

## Rule 2: Understand the limits of your design with regard to your claims

Our scientific claims are only as good as the methods we use to test them, and the research designs used should be appropriate for our research hypotheses, or else they might support the wrong conclusions. This is, of course, true for any and all empirical research. However, inaccurate conclusions are problematic in studies that make socially impactful claims (and especially ones that can affect minoritized social groups). In studies that fall outside the public’s attention, even serious methodological limitations may be acceptable as long as they are clearly addressed. However, when a study reaches public attention, a paragraph summarizing limitations may not be sufficient to curb the study’s potentially negative impact. Often, the general public pays little attention to methodological minutiae. Instead, there is an implicit trust that studies can be interpreted and generalized based solely on their title, abstract, or press release (see [47,68]).

Experts are also not immune from adopting conclusions based on insensitive generalizations, sometimes leading to grievous consequences. For example, autism spectrum disorder (ASD), a complex neurodevelopmental condition often characterized by “persistent deficits in social communication and social interaction across multiple contexts…” [69], has been initially diagnosed as affecting predominantly males, with an estimated male:female ratio ranging from 4:1 to 10:1 (reviewed in [70]). These conceptions have even led scientists to characterize autism as a result of an “extreme male brain,” with females enjoying a “protective” factor ([71]; such notions extend as far back as the 1940s, reviewed in [72]). However, recent research indicates that ASD is underdiagnosed in females and that earlier estimates of the prevalence of the condition included (then unbeknownst) biased samples and diagnostic criteria [70,73–75]. As a result, the prominence of the male brain theory may have severely disadvantaged autistic girls and women, who were underserved by mental health institutions [76] and mistreated by their social environments who maintained the stereotype that autism is a male-only condition [74].

Insensitive generalizations occur across many scientific disciplines, of course, including biology (e.g., almost exclusive reliance on male animal models in inferring population-level effects; [77]), computer science (e.g., face detection algorithms constructed based on almost exclusively white samples; [63,78]), medicine (e.g., treating cisgender men as representative of the human race as far as pathophysiology and treatment of disease go; [79]), psychology (e.g., marking implicit measures of associations as the main target of diversity-training programs; [80]), and others. Therefore, we recommend that researchers whose research touches on social issues (broadly defined) ask themselves earnestly, prior to data collection, what kind of generalization can be made based on their available tools, research design, and the kind of data they can collect. These questions can push us to improve our design, focus our energy on improving our methods, and sharpen the level of generalization appropriate for our findings.

## Rule 3: Incorporate underlying social theory and historical contexts

While the laws of nature are oblivious to our current theories in physics or biology, society and human behavior can be shaped by social theories [31,81]. Throughout history, social policies and hierarchies were justified by the scientific understanding of that time. The resulting social structures then give the semblance of confirming the social theories that shaped them in the first place. In such a reality, merely reporting empirical information without addressing the social structures underlying these data can lead uninformed readers to the wrong conclusions (e.g., [82–84]). Therefore, we suggest that to be more socially responsible, scientists need to take into account the social context both at the design stage (e.g., including measures that can illuminate the role of social context) and as an integral part of our communication efforts. This is especially true for studies documenting between-group differences and studies with clear implications for future social policies.

For example, it is well documented that there are average differences in test scores between different racialized groups in the US [85–87]. Some understood these findings as indicators of stable racial differences rooted in biology, a conclusion that fuels pernicious stereotypes and can cause harm to the stereotyped groups. Moreover, proponents of this view used these findings to promote social policies of diverting funds from students and families from marginalized backgrounds (e.g., [88]). In contrast, many commentators have noted that “race” is not a meaningful biological category (e.g., [82,89,90]) and that test results should be understood in the context of historical structural differences and systemic racism that created education and environmental disparities between various marginalized groups ([87]; see [91] for a variety of views). From this, it follows that more (not less) investment is needed to curb the influence of the social context that created these differences in the first place. Note that reporting on observed differences between groups is not necessarily problematic ([90]) and can even be the first step in creating social policies to address these differences. However, to avoid promoting the wrong conclusions, we should not ignore the myriad of conclusions that can possibly be supported by these results and should make an effort to contextualize them accordingly [31,52]. In such cases, we should incorporate the context as an integral part of the narrative of communicating the findings and not merely as a paragraph summarizing the limitations of the study that will naturally fall outside of public attention.

## Rule 4: Be transparent about your hypothesis and analyses

Every empirical report runs the risk of disseminating findings that eventually turn out to be false. Research shows that motivated reasoning can further increase this risk by leading scientists to conduct and report their analyses in ways that procedurally exacerbate false positives [92,93]. These include, for example, deciding on additional data collection based on obtained results, reporting only results that support a specific narrative, and sequentially conducting multiple analyses until the desired results are obtained. Increased awareness of such risks in recent years has resulted in more and more calls for transparency in the scientific process, including calls for preregistering study protocols and analysis. Preregistration involves developing a comprehensive study protocol that details the hypotheses to be tested, the procedures to obtain the relevant data, and the methods and analyses to test the hypotheses. Although these protocols vary by discipline, an important feature is that they are typically time-stamped. Obtaining a time-stamped registration of the study protocol clearly delineates planned versus post hoc decisions. Even though preregistration can come with certain costs and is not a panacea for all potential problems involved in conducting research [94,95], detailing the planned analyses in advance can safeguard against potential biases that might permeate data collection and analyses, especially in studies where researchers have many degrees of freedom.

In addition, preregistration can inspire confidence in the veracity of one’s analyses. This may be particularly important in studies with meaningful social implications, which are often fervently and critically debated after the fact. Preregistration can curtail any suggestion that the results are only obtained due to post hoc decisions to conduct specific analyses, include or exclude particular variables, or control for certain variables. A potentially even more beneficial form of registration is available in the registered report format, now offered by more than 300 journals in numerous scientific disciplines, ranging from several nature-group journals to discipline-specific ones (e.g., Cochrane Reviews and BMC medicine, Psychological Science, Academy of Management Discoveries) [96]. Registered reports allow scientists to receive peer review on their planned study—before conducting it—and potentially to be conditionally accepted for full publication regardless of the obtained results. Thus, registered reports allow advantages even compared to peer-reviewed research proposals in that they allow publication regardless of specific outcomes (for a practical guideline, see the 10 simple rules by [97]). Notably, (pre-) registration offers transparency mostly for confirmatory hypothesis testing; exploratory analyses remain a critical scientific practice that provides valuable contributions. Here, we emphasize the ability of the registration procedure to guard against the tendency not to report some results of both positive and negative outcomes (the file-drawer problem), a phenomenon that can be particularly problematic in the context of contentious scientific debates that can significantly impact underrepresented groups via public discourse.

## Rule 5: Report your results and limitations accurately and transparently

Publishing an article in a prestigious journal can be an important stepping stone in a scientist’s career. However, these journals typically prioritize simple-to-understand articles that tout substantial theoretical innovation and practical contribution [43]. This means that, even if we are cognizant of the study’s limitation during the design stage (Rule 2), we are still incentivized to simplify, overstate, and sensationalize the impact of our results after we obtain the data. Overstating the implication of our studies can also result in various undesirable outcomes, from allocating public funds to inefficient interventions (e.g., [98]) to skewing public discourse and reducing trust in science in general. One step we can take to curb such negative impacts is to accurately report the limitations of the methodology and our results, including those incompatible with a simplified narrative. Another way to increase both our own and the scientific community’s certainty about the accuracy of our results is to upload our data and analysis procedure to an online repository. This allows other scientists to double-check and reproduce our work, which can reveal difficult-to-detect errors or incorrect inferences. We also recommend ensuring that the data comply with FAIR practices [99] to increase transparency, reproducibility, and reusability.

Of course, an accurate report of results and limitations is a core tenant of any scientific enterprise. However, the possible negative impact of an oversimplified and overstated finding with socially important implications should encourage us to seriously think about limitations that we did not consider at earlier stages of the study. For example, acknowledging possible heterogeneity in samples and results is one way to avoid oversimplification [100,101]. Although a single study can never account for the various ways in which heterogeneity can limit our conclusions, addressing heterogeneity can encourage more incremental scientific progress on this topic and provide a more nuanced understanding to the public and policymakers. More broadly, describing our findings in a manner that closely reflects the obtained results without overselling them can reduce potential misinterpretations and safeguard from problematic usage of one’s findings.

## Rule 6: Choose your terminology carefully

Specialized terminology can have much utility in scientific inquiry by condensing specific concepts and constructs into concise verbal units. However, such specialized terminology can also cause problems when used in a way that seems neutral to some but can carry value-laden connotations for others. Using such loaded terms affects what information people take away from our writing. For example, research on medical terminology has shown that referring to “gout” as “urate crystal arthritis” better aligns participants’ understanding of the disease with contemporary scientific understanding [102].

Choice of terminology may be particularly important when we talk about marginalized groups. In such cases, certain terms can carry connotations related to social stereotypes or core aspects of individuals’ identities. By using such terms, we may be perceived as endorsing stereotypical beliefs and negative views about the marginalized group and may cause stress and genuine hurt to its members [103]. For example, some terminology used when referring to transgender people has been criticized for its implied meaning. Notably, a study coined the term “rapid-onset gender dysphoria” [17] to describe parents’ perceptions regarding changes in their children’s gender identity and expression. In addition to using a term that may mislead others into thinking it represents an established diagnosis, the study was heavily criticized [104–106] for using medical-sounding language such as “cluster outbreaks of gender dysphoria” and “social and peer contagion” that imply that transgender status is tantamount to an infectious disease. Such a conclusion has no empirical support [107] but could nevertheless adversely impact how parents treat their transgender children. More subtly, the often-used terms “transgendered,” “male-to-female” (MTF), or “female-to-male” (FTM) have been criticized for implying that a person “changes” their gender (or has their gender changed by others) rather than changing how other people perceive their gender through coming out [108]. Such implications can be avoided by using terms like “transgender” and “assigned female\male at birth,” which focus on social perception rather than implying essential changes.

Importantly, diversity among people from the same group means that some will prefer terms deemed offensive by others (for example, some transgender people use MTF and FTM to describe themselves). As such, it is possible that a single term can never satisfy everyone. This problem is compounded by the ever-changing nature of language and its shared understanding (e.g., [109,110]). Nevertheless, we should strive to understand the connotations that others associate with our chosen terminology so that we can make educated decisions and minimize harm. We should investigate whether, at a given moment, affected communities have existing best practices when referring to relevant concepts. In qualitative research, this is often achieved by the practice of “member checks” [111], whereby participants are given the opportunity to review, comment, and correct transcripts of interviews and even drafts of the research report. In quantitative research, member checks are often impossible due to the anonymization of participant data. Nevertheless, scientists using quantitative methods can draw on the expertise of stakeholders and advocacy organizations to provide feedback on their use of language. This is especially important when we coin a new term, which is ideally done in collaboration with members of affected groups.

## Rule 7: Seek a rigorous review and editorial processes

A rigorous review—a review that is unbiased, thorough, and follows best reviewing practices [112–114]—is the last line of defense in keeping the scientific literature free from errors and flaws that the authors overlooked. In its ideal form, a rigorous review process involves several knowledgeable peers carefully reviewing the scientific product at hand and providing constructive comments, as well as a careful editor that selects the reviewers, integrates the reviews, and assures the quality of the process. This is especially important for potentially impactful studies for which the bulk of scrutiny often occurs after publication. Therefore, it is also in our best interest to go through a rigorous peer review. A rigorous review also increases the confidence of the research community and the general public in the credibility of the published study and its results. In contrast, unsound editorial practices can result in detrimental outcomes for the original authors and the public sphere alike [60,115]. Although most review processes remain undisclosed, evidence of a rigorous review can be crucial if an article ever comes under public scrutiny. Therefore, we recommend authors submit papers to journals that are reputable for their rigorous process and avoid publishing socially impactful studies in any format that jeopardizes the review process, such as non-peer-reviewed publications or journals that overlook critical points from reviewers (for example, see the publicly available reviewer’s comments for [11], raising much of the concerns that indeed arose after publication). These recommendations also extend to suggesting potential reviewers during submission. Although researchers can use this option to nominate reviewers they think will be favorable to their research [116], the socially responsible approach would be to nominate experts that are likely to be reasonably critical of the study and have with a track record of considering these issues.

Finally, if the manuscript covers a potentially impactful topic, we can alert the editor to this in the cover letter and request extra diligence in the review and the editorial process. In such cases, editors may opt to invite commentaries on the accepted manuscript from opposing researchers [117]. However, in our opinion, such commentaries are not a substitute for a rigorous review, as invested parties often ignore commentaries altogether, even if they point out major flaws in the original paper. For example, Spitzer [118] notoriously claimed to show evidence in favor of the efficacy of “conversion therapy” in changing non-heterosexual orientations. Instead of insisting on a rigorous review process, the editor opted to invite numerous critical commentaries to accompany the paper. Unfortunately, the many flaws detailed by these commentaries did nothing to dissuade organizations that promote conversion therapies from using Spitzer’s article as evidence for their pseudoscientific claims and harmful practices. Spitzer later acknowledged that his paper was flawed and apologized to the gay community for the harm it had caused [119]. With the benefit of hindsight, we now know that such harm would have had higher chances of being avoided altogether if the manuscript had been rigorously reviewed (see also [60] for a discussion of the review and editorial processes that limit racial diversity).

## Rule 8: Play an active role in ensuring correct interpretations of your results

A study can substantially impact public discourse if its conclusions are disseminated through news and social media. To appeal to a broad audience, press releases tend to simplify or sensationalize research findings. Traditional and social media outlets may further amplify this tendency, thus undermining the researchers’ efforts to disseminate their findings responsibly and accurately. Case in point, researchers found that men treat women’s orgasms as an achievement that reaffirms their masculinity [120]. In the article, the authors emphasized that this attitude has negative implications for men and (especially) for women. In contrast, some media outlets reported that the study shows that women’s orgasms benefit men, missing the point entirely. Undoubtedly, some studies can lend themselves more easily to inaccurate interpretations and erroneous narratives than others; however, this example goes to show that even a clearly spelled-out message can be widely misinterpreted.

Naturally, we cannot anticipate all the ways in which our findings can be portrayed or misrepresented. However, to mitigate the impact of these issues, we can be active in how our research is disseminated. For example, in response to the inaccurate article, the authors wrote a press release that further emphasized the negative implications of their findings and sent these to journalists they felt would more accurately report their research, and succeeded in eliciting more accurate coverage [121]. Notably, most academic institutions house public relations offices that can assist in drafting and disseminating such press releases. Scientists can collaborate in drafting a release that accurately reflects scientific findings in a manner accessible to the general public and disseminate it after acceptance but before the study is available online. Although the public relations office may also tend to oversimplify the results, it is much easier to influence and sharpen the university’s press release than to influence news outlets’ reporting. Furthermore, we can track the impact of our studies via tools such as Altmetric and follow up with prominent media outlets to ask for corrections. If such requests are refused or ignored, we can report the inaccuracies to independent regulators who can force corrections (e.g., the Independent Press Standards Organisation in the United Kingdom). We can also engage in social and traditional media discussions with the help of media professionals from our institutions. This can take the form of social media posts, replies, quotes, and interviews for traditional media. In sum, although not in our typical scientific skill set (and as such, more difficult to contend with), there are ways for us as researchers who are interested in responsible dissemination of our findings to actively engage in the impact of our research in the public sphere and ensure the public is exposed to more accurate accounts of our findings and their implications.

## Rule 9: Address criticism from peers and the general public with respect

Studies that touch on socially contentious issues or other identity-related topics will often result in heated responses from peers and communities affected by this work. Online platforms that incentivize quick responses and engagement, like Twitter, can exacerbate these responses and create self-reinforcing cycles that accentuate polarized interpretations of specific findings. Even if started in good faith, such online discussions might devolve quickly into bitter moral arguments between opposing camps. In such arguments, the most harm is often inflicted on the more vulnerable members of the community, be it early career researchers, individuals from marginalized backgrounds, or any other potential vulnerability.

Despite the very emotional (and often personal) nature of these discussions and their rapid deterioration, it is important that we do not rush to respond. The sheer volume of negative responses can be overwhelming, and treating all commentators as a single group is tempting. However, some adversarial claims will have substantive criticism that we will be able to refute. Some substantive claims will offer new insights or potential limitations we did not consider in advance. Yet other claims might express genuine hurt, especially in cases where individuals feel that our findings and conclusions affect a core aspect of their identity. Differentiating these points can be very difficult in the heat of the moment. Nevertheless, we suggest that it is best if we address substantive criticisms with respect and address the unintended harm our research might have caused, keeping in mind potential limitations in our perspective and our study.

## Rule 10: When all else fails, consider submitting a correction or a self-retraction

Despite the best of intentions, we might realize only after publication that our article has harmful implications or is otherwise flawed. This occurs more often when we are open to learning new things about the subject matter from any critical comments we receive after publication. If we change our minds and become convinced that our publication is flawed, we might consider issuing a correction or retracting the paper altogether. A correction can be issued to alert the readers about flaws that do not take away from the main point of the article. In contrast, a retraction may be in order when the flaw relates to a key measure, analysis, or conclusion. For example, a recent study [122] about the potential benefits of hydroxychloroquine for treating Coronavirus Disease 2019 (COVID-19) was retracted by some of the authors because they could no longer stand behind “the veracity of the primary data sources” [123]. Whether hydroxychloroquine helps treat COVID-19 or not, studies that present support for an ineffective treatment can result in catastrophic consequences. Due to media attention, new (and potentially ineffectual or harmful) COVID-19 treatments were broadly (and prematurely) adopted by medical staff in numerous clinics around the world. A retraction suggests that the scientific establishment, and in this case, the authors themselves, have lost confidence in the study, which can be used to argue against the premature adoption of these conclusions.

There are good reasons why we may consider self-retracting a majorly flawed article. First, retractions are the ultimate tool to correct the public record, as they alert readers that a study should not be relied upon (but see [124] for potential issues even with retractions). Retractions are important because policymakers, interested parties, and other researchers may still rely on the original flawed article, even if the authors disavow their own conclusions in a subsequent publication. Moreover, retracting a potentially harmful study signals to the public and other scientists that the authors, in particular, and scientists, in general, take the responsibilities given to them seriously. If we decide to retract a paper, the best course of action is to discuss this with the editor and write a detailed notice explaining the reasons that led us to retraction. Finally, despite the cost to authors incurred by retractions, self-retractions can be beneficial, especially when compared to journal-initiated retractions. Journal-initiated retractions are often taken as an indication of wrongdoing, even when no malfeasance took place. In contrast, authors who self-retract may be lauded as “heroic” [125,126] for admitting an error and being willing to sacrifice a publication for the greater good. If we become convinced that our paper promotes harm, it is better to be remembered as the person who courageously admitted a mistake than the authors of a socially harmful paper.

### Summary

Communicating one’s scientific findings to peers and the general public is integral to the scientific endeavor. Without informing our discipline about our important results, theories cannot be updated, and knowledge cannot be accumulated. Likewise, disseminating our findings to the public and policymakers can shape public discourse and encourage the implementation of more scientifically accurate policies. However, due to a lack of training and structural support, scientists may be unaware of the potential social impact of their findings. For example, an artificial intelligence expert might build an excellent new generative language model but may unintentionally overlook their model’s bias when it comes to indigenous populations. Unfortunately, once a specific finding with a particular interpretation has gained public traction, updating or correcting the interpretation requires significant efforts that often fail (e.g., the impact of an infamous study on vaccine skepticism; [127,128]).

Should such potential implications dissuade researchers from conducting socially impactful research? As scientists, we believe that scientific and social progress hinges on searching for empirical truths and better theories and that potential misuse of a scientific study should typically not provide sufficient grounds for not publishing or conducting it in the first place. However, we also believe that social responsibility and scientific merit are not diametrically opposed. Therefore, in the spirit of the recent push towards more active engagement with the social impact of scientific research (e.g., [47,52,129–131]), we suggested 10 simple rules to help scientists consider socially responsible aspects of their work. By following these suggestions, we believe that scientists will be better able to foresee and minimize potential harms and, at the very least, be better prepared for post-publication discussions related to their research.

We recognize that these recommendations work, at times, against the authors’ incentives and are not a substitute for structural change in how scientific research is conducted and rewarded. This conflict of interest between publishing socially responsible science and the authors’ incentives is especially harsh for early career researchers who need publications in prestigious journals to get a permanent job. Therefore, we call on scientific societies, research institutions, and funding agencies to take active steps to encourage and reward social responsibility. Given the broader societal implications and the unintended harm that has already been caused time and again, we believe there is no better time than the present to start engaging with this important topic.
